# How ATP-Dependent Chromatin Remodeling Complexes Regulate Vertebrate Embryonic Development

**DOI:** 10.3390/ijms27020835

**Published:** 2026-01-14

**Authors:** Hejie Wang, Gulinigaer Anwaier, Shengbin Bai, Libin Liao, Yingdi Wang, Shuang Li

**Affiliations:** 1 Department of Histology and Embryology, School of Basic Medical Sciences, Xinjiang Medical University, Urumqi 830017, Xinjiang, China; wanghejie@stu.xjmu.edu.cn (H.W.); baishengbin@xjmu.edu.cn (S.B.); liaolb@xjmu.edu.cn (L.L.); wangyingdi@stu.xjmu.edu.cn (Y.W.); 2 Department of Pathophysiology, School of Basic Medical Sciences, Xinjiang Medical University, Urumqi 830017, Xinjiang, China; gulinigaer@xjmu.edu.cn

**Keywords:** embryonic development, chromatin remodeling complexes, embryonic genome activation, lineage specification, epigenetic modifications

## Abstract

ATP-dependent chromatin remodeling complexes regulate gene expression by altering chromatin structure through ATP hydrolysis. They are classified into four families—SWI/SNF, ISWI, CHD, and INO80—which remodel chromatin via nucleosome sliding, eviction, assembly, and editing to control transcription. These complexes play critical roles in DNA repair, tumorigenesis, and organogenesis. Recent advances in low-input proteomics have highlighted their importance in vertebrate embryonic development. In mammals, they regulate embryonic genome activation, lineage specification, and stem cell fate determination. In non-mammalian models (e.g., *Xenopus laevis*), they function from blastocyst formation to pre-organogenesis stages (gastrulation and neurulation)—key windows for chromatin reprogramming and cell fate decisions. This review provides a systematic overview of chromatin remodeling complexes, detailing their classification and conserved mechanisms, and discusses their functions in early embryogenesis and embryonic stem cell maintenance. The collective evidence underscores the implications of these chromatin remodelers for understanding developmental defects and advancing regenerative medicine.

## 1. Introduction

Embryonic development is a highly regulated process that depends on precise spatiotemporal regulation of gene expression. This regulatory machinery is inextricably linked to dynamic chromatin structural remodeling. As the primary carrier of genetic information, chromatin undergoes extensive reorganization during embryogenesis, dynamically modulating DNA accessibility and transcriptional activity [1]. The transition from transcriptionally quiescent zygotes to pluripotent blastocysts, followed by lineage commitment and tissue morphogenesis, relies on the intricate coordination between chromatin accessibility landscapes and gene regulatory networks. While transcription factors have long been regarded as central regulators of developmental programs [2,3,4], emerging research underscores the pivotal role of ATP-dependent chromatin remodeling complexes. These molecules regulate chromatin structure to modulate DNA accessibility and transcription during embryonic development [5,6].

Despite significant advances in developmental biology, current research predominantly focuses on transcription factor-centric mechanisms, revealing substantial gaps in our systematic understanding of chromatin remodelers. Powered by ATP hydrolysis, these complexes dynamically sculpt the chromatin landscape through nucleosome repositioning, ejection, and histone variant exchange [7,8]. Experimental studies in mammalian embryos and embryonic stem cells indicate that these complexes are required for key developmental events, including embryonic genome activation and lineage specification [5,9]. Accumulating evidence indicates that chromatin accessibility and transcriptional competence are progressively established during preimplantation development, highlighting the essential role of early chromatin remodeling in enabling zygotic genome activation (ZGA) and cell fate determination [10,11]. However, the precise molecular mechanisms by which distinct remodeling families regulate chromatin dynamics in vivo during embryogenesis remain poorly defined. In addition, although mutations in several remodeler subunits are linked to develop-mental disorders, the direct mechanisms connecting remodeler dysfunction to specific embryonic phenotypes are still unclear [5,12,13,14,15].

This review aims to summarize current knowledge on ATP-dependent chromatin remodeling complexes in embryonic development. We classify these complexes based on their structural and functional features and discuss experimental evidence supporting their roles in embryonic genome activation, lineage specification, and embryonic stem cell fate determination. Where possible, we draw on cross-species comparisons to distinguish conserved principles from species-specific regulatory features. Rather than proposing definitive mechanistic models, this review focuses on several unresolved questions that emerge from the current literature, including: (1) how different remodeling families may act sequentially or in parallel during key developmental transitions; (2) how chromatin remodelers interact with transcription factor networks to influence cell fate decisions; and (3) how species-specific timing and expression patterns of remodelers may shape developmental outcomes. By integrating transcriptomic, proteomic, and functional studies across model systems, we aim to clarify what is currently supported by experimental evidence, identify major knowledge gaps, and outline directions for future mechanistic investigation.

## 2. Classification and Function of Chromatin Remodeling Families

### 2.1. Overview of Chromatin Remodeling Complex Families

**Figure 1:**
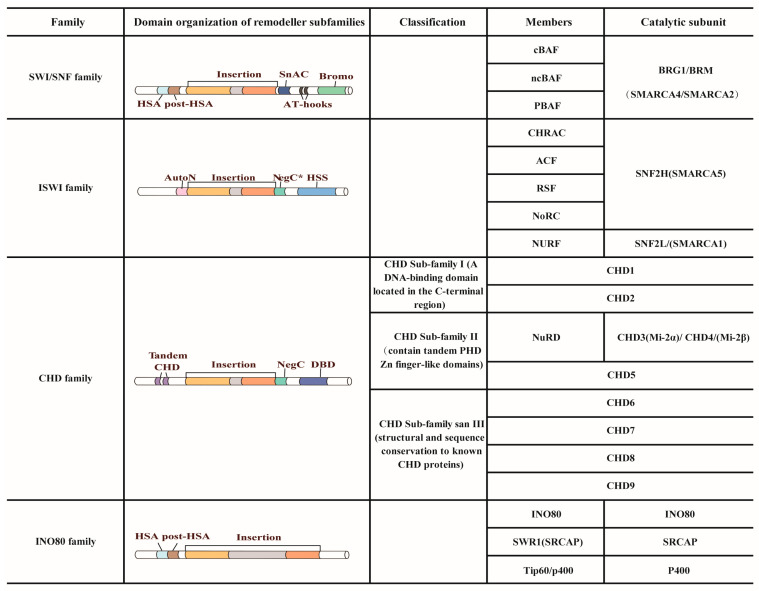
Classification of ATP-dependent chromatin remodeling complex families. NegC, negative regulator of coupling; NegC*, a region structurally similar to the ISWI NegC domain; Bromo, bromodomain; DBD, DNA-binding domain; AutoN, autoinhibition N-terminal region; HSS, HAND–SANT–SLIDE domain; HSA, helicase-SANT-associated domain; SnAC, Snf2 (structural maintenance of chromosomes protein 2) ATP coupling domain; Tandem CHD, Tandem chromodomain; AT-hook, AT-hook-like DNA-binding motif.

Chromatin is organized into a condensed nucleoprotein structure that limits the access of transcription factors to DNA and thereby influences gene expression [16]. Chromatin remodeling involves the ATP-dependent dynamic reorganization of chromatin architecture, which modulates DNA accessibility. This process facilitates the binding of transcription factors and other regulatory proteins, ultimately controlling gene transcription and other DNA-dependent processes [17]. Chromatin remodeling is achieved in two main ways: ATP-independent chromatin remodeling, mainly through histone modifications, such as acetylation, methylation or phosphorylation of lysine, arginine and other residues in histone tails [18,19]. These modifications regulate chromatin structure and function by altering histone-DNA interactions or recruiting effector proteins. Another type of chromatin remodeling is ATP-dependent chromatin remodeling, in which the energy generated by ATP hydrolysis is used to reposition, evict, or restructure nucleosomes, thereby altering the relative organization of histones and DNA. A variety of ATP-dependent chromatin remodeling complexes are involved in this energy-dependent chromatin remodeling process [8,20]. ATP-dependent chromatin remodeling complexes are multifunctional protein complexes consisting of a core subunit and multiple accessory subunits [21,22]. Depending on the structural domains of the core subunits of ATPases, chromatin remodeling complexes are classified into four major families: sucrose non-fermentable variants (SWI/SNF), imitation switches (ISWI), chromodomain helicase DNA-binding proteins (CHD) and inositol requiring factor 80 (INO80). These family complexes differ significantly in structure and function and exhibit different biochemical activities [23,24] (Figure 1).

#### 2.1.1. SWI/SNF Family

The SWI/SNF complex was first identified in the 1980s in yeast and subsequently in mammalian systems in the 1990s [25]. In mammals, the family can be divided into three subtypes based on subunit composition: canonical BRG1 (Brahma-related gene 1)/BRM (Brahma)-associated factor (cBAF), polybromo-associated BAF (PBAF), and non-canonical BAF (ncBAF, also called GBAF) [26,27]. These complexes share the ATPase catalytic subunits BRG1/BRM (also known as SMARCA4/SMARCA2) and the core subunits BAF155/BAF170 (also known as SMARCC1/SMARCC2), and use ATP hydrolysis-driven nucleosome reconfiguration to regulate chromatin accessibility [26]. Numerous studies have shown that the SWI/SNF complex plays a role in regulating several key biological processes, including embryonic development, tissue regeneration, and cancer progression [28,29,30].

#### 2.1.2. ISWI Family

Members of the ISWI family, also referred to as the SNF2H/SNF2L family in mammals, include nucleosome-remodeling factor (NURF) [31], chromatin accessibility assembly complex (CHRAC) [32], ATP-utilizing chromatin assembly remodeling factor (ACF) [33], nucleolar remodeling complex (NoRC) and remodeling structure of chromatin factor (RSF) [34]. These complexes share catalytic ATPase subunits SNF2H (Sucrose Non-Fermenting 2 Homolog H, also called SMARCA5) and SNF2L (Sucrose Non-Fermenting 2 Homolog L, also called SMARCA1), together with two to four auxiliary subunits, which contribute to gene transcription, DNA replication and repair, and the maintenance of chromatin homeostasis during embryonic development [35,36,37].

#### 2.1.3. CHD Family

Members of the CHD family participate in the regulation of chromatin dynamics, altering nucleosome structure and DNA accessibility [12]. The characteristic structure of CHD family proteins lies in the fact that their N-terminal domain contains two tandemly arranged chromodomains, which specifically recognize and bind methylated histones [38]. This family can be divided into three subfamilies: subfamily I (CHD1/CHD2), subfamily II (CHD3–5), and subfamily III (CHD6–9) [8]. The CHD family members exist as monomers or multimeric complexes. CHD3/CHD4 (Mi-2α/β) interact with histone deacetylases (HDAC1/2) to form the nucleosome remodeling and deacetylase (NuRD) complex. This multifunctional complex plays a key regulatory role in processes such as embryonic development, cell differentiation and tumor formation [39,40,41].

#### 2.1.4. INO80 Family

The INO80 family includes SWI2/SNF2-related 1 (SWR1), IN080, Tat-interacting protein 60 kDa (TIP60) complex, or E1A binding protein p400 (EP400) complex. The catalytic subunit of this family contains a characteristic insertion sequence between the structural domains of the ATPase, which recruits cofactors such as the deconjugating enzyme RuvB-like AAA ATPase 1/2 (RUVBL1/2) and provides a scaffold for additional cofactor binding [42]. The INO80 chromatin remodeler has been implicated in the regulation of gene expression, DNA replication, and DNA damage response in multiple experimental systems [43,44,45].

### 2.2. Core Mechanisms of Gene Expression Regulation by Chromatin Remodeling Complexes

**Figure 2:**
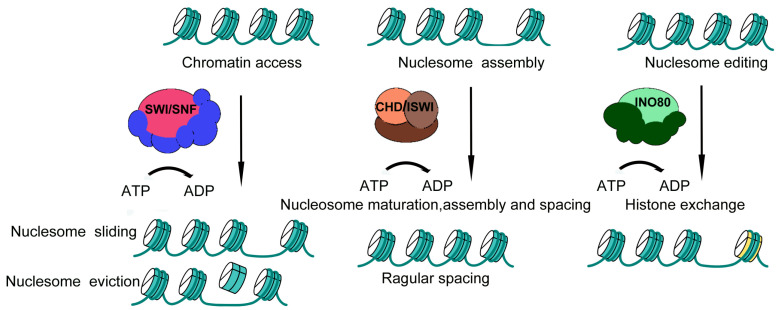
Three models of how chromatin remodeling factors regulate gene expression. ISWI and CHD family remodelers primarily reposition nucleosomes via assembly-coupled mechanisms to regulate gene expression, while SWI/SNF and INO80 complexes mediate chromatin remodeling through nucleosome sliding/eviction and nucleosome editing, respectively. Nucleosome editing (histone variant exchange) is depicted in yellow.

Chromatin remodeling complexes dynamically regulate the affinity between histones and DNA to change the structure of chromatin. Through these mechanisms, chromatin remodeling complexes regulate gene expression without altering the underlying DNA sequence, representing a key layer of epigenetic control [46]. Its main modes of regulation include nucleosome sliding, nucleosome eviction, nucleosome assembly, and nucleosome editing (Figure 2). Different families regulate gene expression in different ways: the SWI/SNF family is mainly involved in the regulation of chromatin accessibility through sliding and eviction of nucleosomes [47]; the ISWI and CHD families are mainly involved in the regulation of nucleosome assembly [47,48]; and the INO80 family achieves nucleosome editing by exchanging histone variants [49]. All of these mechanisms alter DNA accessibility, thereby affecting specific physiological processes [9,50].

#### 2.2.1. Nucleosome Sliding and Eviction

Nucleosome sliding and eviction refer to ATP-dependent processes in which chromatin remodeling complexes reposition or remove nucleosomes to expose regulatory DNA elements. These activities facilitate access to promoters, enhancers, and other functional genomic regions required for transcription, DNA repair, or recombination [5,8]. These mechanisms are best characterized for the SWI/SNF family remodelers, which use ATP hydrolysis to reposition nucleosomes and increase chromatin accessibility at regulatory elements such as promoters or enhancers [8,51]. Nucleosome eviction occurs when SWI/SNF family members create nucleosome-depleted regions (NDRs) or nucleosome-free regions (NFRs) at gene promoters, establishing favorable conditions for transcription initiation [52,53].

#### 2.2.2. Nucleosome Assembly

Nucleosome assembly involves the ATP-dependent remodeling of pre-nucleosomes into mature histone octamers wrapped by 146 bp DNA, followed by the establishment of evenly spaced nucleosome arrays. This process contributes to chromatin compaction and gene silencing, particularly in heterochromatic regions [54]. Members of the ISWI and CHD family complexes specifically bind linker DNA and regulate nucleosome spacing through conserved HAND–SANT–SLIDE (HSS) structural domains, thereby ensuring orderly nucleosome array organization [55]. For example, ISW1a maintains yeast nucleosome array alignment by sensing the chromatin microenvironment [56], whereas CHD4 represses gene expression by controlling nucleosome positioning to limit transcription factor access [57].

#### 2.2.3. Nucleosome Editing

Nucleosome editing is an ATP-dependent process dominated by the INO80 family of chromatin remodeling complexes, which remodel chromatin accessibility and directionally regulate gene expression by exchanging histone variants [8,54]. This process can both activate and repress transcription [8]. Core members of the INO80 family, including the INO80 complex and the SWR1 complex (also known as SRCAP in mammals), catalyze the replacement of canonical histones with variant histones [10,58]. Notably, the INO80 chromatin remodeler catalyzes the replacement of canonical H2A with the H2A.Z variant in nucleosomes in vitro, driving nucleosome turnover [59,60,61]. The SWR1 complex specifically catalyzes the replacement of H2A-H2B dimers with H2A.Z-H2B dimers in nucleosomes [62]. This dynamic nucleosome editing modulates local chromatin structure, recruits chromatin-modifying factors, and maintains chromatin in open or repressed states, thereby coordinating the balance between gene activation and silencing [54].

## 3. Roles of Chromatin Remodeling Factors in Mammalian Early Embryonic Development

**Figure 3:**
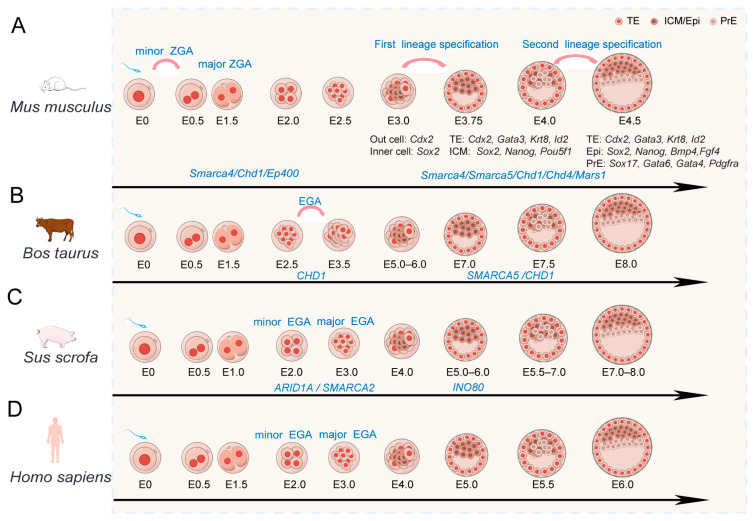
Research on the functions of chromatin remodeling factors during early embryonic development in different species. (**A**) During early embryonic development in the mouse (*Mus musculus*) from E0 to E4.5, functional studies indicate roles for *Smarca4*, *Chd1*, and *Ep400* in ZGA, and *Smarca4*, *Smarca5*, *Chd1*, *Chd4*, and *Mcrs1* in lineage specification. (**B**) During early embryonic development in bovine (*Bos taurus*) from E0 to E8.0. *CHD1* is implicated in EGA, while *SMARCA5* and *CHD1* function in lineage specification. (**C**) During early embryonic development porcine (*Sus scrofa*) from E0 to E8.0. *ARID1A* and *SMARCA2* function during EGA, and *INO80* is involved in lineage specification. (**D**) During early embryonic development human (*Homo sapiens*) from E0 to E6.0. Developmental timing is like porcine, but functional evidence for chromatin remodelers is currently lacking. E, embryonic day; TE, trophectoderm; ICM, inner cell mass; Epi, epiblast; PrE, primitive endoderm.

During mammalian early embryogenesis, a highly conserved developmental program orchestrates a cascade of precisely regulated events, encompassing embryonic genome activation (EGA), compaction, polarization, and lineage specification, ultimately leading to blastocyst formation [63,64] (Figure 3). As a pivotal event initiating early embryogenesis, EGA marks the transition from maternal transcript dominance to zygotic genome-driven gene expression [10]. The first lineage specification separates the morula into the inner cell mass (ICM) and the trophectoderm (TE), while the second lineage specification further segregates the ICM into the epiblast (EPI) and primitive endoderm (PE) [63,64]. Proper regulation of these transitions depends on coordinated changes in chromatin structure and gene expression. Increasing evidence indicates that chromatin remodeling factors contribute to these processes during early embryonic development (Table 1).

### 3.1. Chromatin Remodelers in Embryonic Genome Activation (EGA)

#### 3.1.1. Timing and Developmental Context of EGA Across Mammals

During the early stages following fertilization, the embryo primarily relies on maternally provided mRNA and proteins. As the embryo develops further, its genome must be activated to initiate its own developmental program. Embryonic genome activation (EGA) marks the transition of the genome from transcriptional silence at fertilization to an active state [64]. The timing of EGA varies across different species. In mice, EGA occurs from the late 1-cell stage to the mid-late 2-cell stage [10] and is specifically referred to as ZGA (zygotic genome activation). ZGA can be categorized into major ZGA and minor ZGA phases. In other mammals, EGA occurs during the embryonic period after the 2-cell stage, such as at the 4-cell stage in porcine [78], the 8– to 16-cell stage in cattle [79], and the 4– to 8-cell stage in human [80] (Figure 3). These interspecies differences in the timing of EGA have been documented across mammals, although the underlying contributions of chromatin state, maternal factors, and transcription factor availability remain incompletely understood.

#### 3.1.2. Chromatin Priming and Accessibility: The Central Role of SWI/SNF

Chromatin remodelers are essential for initiating early chromatin accessibility required for EGA. The SWI/SNF ATPase BRG1 plays a pivotal role in establishing an open chromatin environment. In mice, depletion of maternal *Brg1*, the catalytic ATPase subunit of SWI/SNF complexes, causes developmental arrest at the two-cell stage and impairs transcription of approximately 30% of zygotic genes [14]. These observations suggest that SWI/SNF activity is required for proper chromatin accessibility during EGA, although the direct genomic targets and sequence of events remain to be defined. Consistently, BRG1 overexpression in donor cells enhances chromatin accessibility and EGA-related transcription in porcine somatic cell nuclear transfer (SCNT) embryos, indicating that SWI/SNF-mediated remodeling facilitates genomic reactivation during nuclear reprogramming [81].

ARID1A, a SWI/SNF subunit, also contributes to early chromatin reorganization. In porcine embryos, ARID1A depletion disrupts embryonic development and is associated with altered histone modification patterns and changes in SWI/SNF complex localization [13]. However, how these changes mechanistically influence EGA remains unclear. Together, these studies indicate that SWI/SNF complexes contribute to chromatin reorganization during early stages of EGA, but the causal relationship between chromatin remodeling and transcriptional activation requires further mechanistic investigation.

#### 3.1.3. Histone Variant Deposition for Transcriptional Initiation: Roles of CHD1 and EP400

The major wave of EGA requires extensive histone variant incorporation and nucleosome remodeling. CHD1 is indispensable for proper ZGA. In mouse embryos, *Chd1* depletion disrupts the activation of key ZGA genes such as *Hmgpi* [73], while in cattle embryos, *CHD1* deletion markedly reduces development to the 8–16-cell stage and blastocyst formation [72]. Although these phenotypes differ across species, both studies support a role for CHD1 in facilitating histone H3.3 deposition during early embryonic transcriptional activation [72,73].

EP400, initially identified as an E1A-binding protein, functions as an H3.3 histone chaperone that facilitates histone variant incorporation to promote transcriptional activation [82,83]. Maternal EP400 deficiency in mice leads to EGA failure and developmental arrest at the 2–4-cell stage, accompanied by reduced H3.3 deposition and impaired transcriptional elongation at ZGA-associated promoters [75]. These findings suggest that EP400 contributes to EGA by supporting histone variant dynamics during early transcriptional activation.

Taken together, available evidence indicates that multiple chromatin remodeling factors contribute to successful EGA. While certain mechanisms, such as histone variant deposition and nucleosome repositioning, appear conserved, the specific regulatory strategies and developmental timing vary across species. Further comparative and mechanistic studies will be required to clarify how these remodelers are integrated into species-specific programs of early embryonic transcription.

### 3.2. Chromatin Remodelers in Lineage Specification

#### 3.2.1. An Overview of Lineage Specification and the Role of Chromatin Remodelers

Lineage specification, a pivotal event in early embryonic development, involves the progressive commitment of pluripotent cells to distinct lineages, a process directed by core transcription factors such as OCT4 (encoded by *Pou5f1*) and CDX2 [84]. These factors maintain pluripotency and initiate differentiation, respectively, through a mutually repressive interaction in mice [2,85,86,87]. However, this reciprocal regulation is not fully evolutionarily conserved, as OCT4 and CDX2 are transiently co-expressed in the trophectoderm of cattle embryos, indicating species-specific regulatory divergence [88]. In addition to transcription factor-mediated control, accumulating evidence highlights that chromatin remodeling factors contribute to the establishment of chromatin states that permit or restrict lineage-specific gene expression during the first lineage specification [11,64]. How these remodelers functionally interact with transcription factor networks during early lineage decisions remains an important but incompletely resolved question.

#### 3.2.2. SWI/SNF Complexes: Partnering with Transcription Factors to Reinforce Lineage Identity

SWI/SNF complexes play a critical role in reinforcing lineage identity by partnering with key transcription factors. In mice, loss of the SWI/SNF ATPase BRG1 disrupts early lineage specification by failing to repress *Nanog* in the trophoblast, as BRG1 cooperates with HDAC1 to mediate histone deacetylation and nucleosome remodeling at the *Nanog* enhancer during preimplantation development [89]. Consistent with this, single-cell CUT&RUN analyses indicate that NANOG chromatin binding in blastocysts depends on intact SWI/SNF activity in vivo [90]. Furthermore, BRG1 cooperates with CDX2 to repress *Oct4* transcription at the blastocyst stage, thereby ensuring proper trophoblast development [65]. Similarly, the T-box transcription factor EOMES has been reported to cooperate with SWI/SNF complexes to maintain chromatin accessibility at trophectoderm-associated loci during implantation [91]. Together, these observations support a role for SWI/SNF complexes in modulating chromatin accessibility at lineage-specific regulatory elements in coordination with transcription factors. However, the extent to which these interactions are direct and their temporal dynamics during lineage commitment remain to be fully defined.

#### 3.2.3. ISWI Complexes: Ensuring Structural Integrity for Lineage Progression

SMARCA5 belongs to the ISWI family of chromatin remodeling factors. As a conserved ISWI family member, SMARCA5 exhibits functional heterogeneity among species. In mice, knockdown of *Smarca5* resulted in decreased blastocyst formation and defective ICM differentiation [70]. In cattle, *SMARCA5* deletion significantly reduced blastocyst quality and the proportion of PE cells, but did not affect the rate of blastocyst development [70]. Therefore, SMARCA5 is required for successful lineage-specific programming in mice and cattle. These observations suggest that SMARCA5 may function to support chromatin structure and transcriptional programs required for lineage progression, although this interpretation will require direct functional validation in vivo.

#### 3.2.4. CHD Family: Maintaining Transcriptional Balance and Fidelity

The CHD family is essential for maintaining the transcriptional balance and fidelity required for lineage segregation. In mouse embryos, *Chd1* knockdown significantly reduces the expression of key lineage-specific transcription factors, including *Cdx2*, *Oct4*, and *Nanog*, and causes embryonic lethality after implantation [73]. In contrast, studies in mouse embryonic stem cells have shown that CHD4 safeguards the fidelity of lineage segregation by restricting premature activation of lineage-specific genes through its remodeling activity [92,93]. In mouse embryos, zygotic *Chd4* knockout increases the frequency of lineage-specific gene expression in unspecified cells, leading to failed trophectoderm formation and implantation failure [74]. However, the contribution of maternal CHD4 remains unclear, as maternal protein persistence after ZGA may partially compensate for zygotic loss in mouse embryos [74]. Together, these findings indicate that CHD1 and CHD4 contribute to lineage specification through distinct mechanisms: CHD1 is required for activating or maintaining key lineage-specific transcriptional programs, while CHD4 restricts inappropriate gene expression to ensure proper lineage segregation.

#### 3.2.5. INO80 Family: Linking Chromatin Remodeling to Cellular Morphogenesis

The INO80 family links chromatin remodeling to the cellular morphogenesis essential for lineage specification. In porcine embryos, INO80 is required for blastocyst development, where it maintains trophectoderm epithelial integrity and regulates genes critical for TE polarity and lumen formation (e.g., *ADAM19*, *CDH1*, *ACTA2*, etc.) [76]. Separately, MCRS1, a factor associated with the INO80 complex, has been recognized as an epiblast (EPI)-specific marker in human blastocysts [94,95]. Deletion of *Mcrs1* in mice markedly reduces EPI cell numbers and disrupts EPI lineage formation [77]. These findings suggest that INO80-associated factors contribute to lineage specification by influencing both transcriptional programs and cellular architecture, although the direct mechanistic links between chromatin remodeling and morphogenetic processes remain to be clarified.

### 3.3. Chromatin Remodelers Govern Stem Cell Differentiation

#### 3.3.1. Stem Cells as a Model and the Dual Role of Remodelers

Mouse embryonic stem cells (mESCs) provide a powerful system to dissect how chromatin remodeling orchestrates the transition from pluripotency to lineage commitment. Chromatin remodelers not only maintain pluripotency gene expression and self-renewal capacity but also reconfigure the epigenetic landscape to prime cells for differentiation [96]. Distinct chromatin remodeling complexes achieve their regulatory specificity through diverse subunit compositions and mechanistic modes, collectively ensuring precise temporal control of pluripotency and lineage gene expression.

#### 3.3.2. SWI/SNF Complexes: Context-Dependent Regulation of Pluripotency and Differentiation

SWI/SNF family remodelers are key context-dependent regulators of ESC fate, with functional diversity arising from combinatorial subunit assembly [97]. The core subunit SMARCC1 (BAF155) represses pluripotency genes such as *Nanog* by promoting heterochromatin formation, and its deletion leads to chromatin decompaction and impaired differentiation [98]. In contrast, the embryonic stem cell–specific BAF (esBAF) complex, characterized by the inclusion of BRG1, BAF155, and BAF60A but exclusion of BRM, BAF170, and BAF60C, maintains pluripotency and prevents premature differentiation [99]. SMARCA4 (BRG1) deficiency compromises the binding of key pluripotency transcription factors (e.g., STAT3, OCT4, SOX2, NANOG) to chromatin [100,101,102]. SMARCB1 prevents lineage mis-specification by maintaining open chromatin at pluripotency loci [103]. These studies indicate that distinct SWI/SNF assemblies exert context-specific effects on chromatin structure and transcription in embryonic stem cells. How these complexes are dynamically regulated during the transition from pluripotency to differentiation remains to be fully elucidated.

#### 3.3.3. SNF2-Family Remodelers: Custodians of Heterochromatin and Epigenetic Stability

A distinct set of SNF2-family ATPases reinforces pluripotency by safeguarding heterochromatin integrity and epigenetic stability. SMARCAD1, a member of this family, interacts with KRAB-associated protein 1 (KAP1) via its CUE1 domain and maintains heterochromatin silencing at KAP1 target loci, including zinc finger protein genes and imprinted regions. This KAP1–SMARCAD1 axis preserves nuclear localization, chromatin binding, and genome stability in ESCs; disruption of this interaction perturbs heterochromatin organization and compromises pluripotency maintenance [104]. Beyond the canonical remodelers, SNF2-like ATPases such as Lymphoid-specific helicase (LSH, also known as HELLS) and Alpha-Thalassemia/Mental Retardation X-linked protein (ATRX) play complementary roles in ESCs. LSH interacts with ten-eleven translocation (TET) proteins and contributes to the regulation of 5-hydroxymethylcytosine (5hmC) levels and genomic distribution in ESCs, as loss of LSH leads to a global reduction and redistribution of 5hmC independent of DNA methylation changes [105]. Meanwhile, ATRX deposits the replication-independent histone variant H3.3 into heterochromatic regions to maintain repressive H3K9me3 marks and epigenetic memory [78]. Together, these observations indicate that SNF2-family remodelers participate in the regulation of heterochromatin structure and epigenetic features associated with pluripotency, although the extent to which these activities directly influence lineage commitment remains to be determined.

#### 3.3.4. CHD Family: Fine-Tuning Lineage Commitment Through Distinct Domains and Complexes

CHD family remodelers fine-tune the balance between pluripotency and commitment through structural domain–specific mechanisms. The N-terminal serine-rich region (SRR) of CHD1 is required for proper lineage differentiation, as SRR deletion induces aberrant lineage specification [106]. In contrast, CHD4 suppresses T-box transcription factor 3 (*Tbx3*) expression and cooperates with the histone variant H2A.Z to coordinate pluripotency gene activation and differentiation gene repression, thereby preserving ESC identity and developmental potential [92]. These findings suggest that CHD1 and CHD4 exert distinct regulatory effects during the exit from pluripotency, although how their activities are coordinated during lineage commitment remains incompletely understood.

#### 3.3.5. INO80 Family: Coupling Chromatin Remodeling to the Core Pluripotency Network

INO80 and related remodelers couple chromatin remodeling with transcriptional activation of the pluripotency network. The INO80 complex occupies promoters of core pluripotency genes alongside OCT4 and WD repeat-containing protein 5 (WDR5), maintaining an open chromatin state and facilitating recruitment of the mediator complex and RNA polymerase II. This mechanism is critical for ESC self-renewal, reprogramming efficiency, and blastocyst development [107]. The related Tip60–p400 complex contributes to pluripotency maintenance through non-catalytic restriction of chromatin accessibility, while its lysine acetyltransferase activity is required for activating mesodermal and endodermal genes during differentiation [108]. Together, these findings indicate that INO80-family complexes participate in the regulation of pluripotency and differentiation through multiple chromatin-associated mechanisms, although their precise roles during lineage commitment require further clarification.

## 4. Systematic Roles of Remodeling Factors Across Mammalian Early Embryogenesis

To complement the functional studies summarized above, we next examined whether publicly available transcriptomic and proteomic datasets exhibit conserved expression patterns of chromatin-remodeler genes across mammalian species. These data were retrieved directly from the original publications, which had already performed full normalization and quality control. In this review, we extracted the expression values of remodeler subunits and visualized their temporal dynamics without additional computational processing, thereby preserving the integrity of the source datasets. For adjacent developmental stages, unpaired two-tailed Student’s *t*-tests were applied following confirmation of variance homogeneity to evaluate relative changes in expression. This integrative analysis provides a comparative molecular context for relating remodeler expression dynamics to known developmental transitions during early embryogenesis.

### 4.1. Cross-Species Transcriptomic Patterns Validate Stage-Specific Functions of Remodeler Families

**Figure 4:**
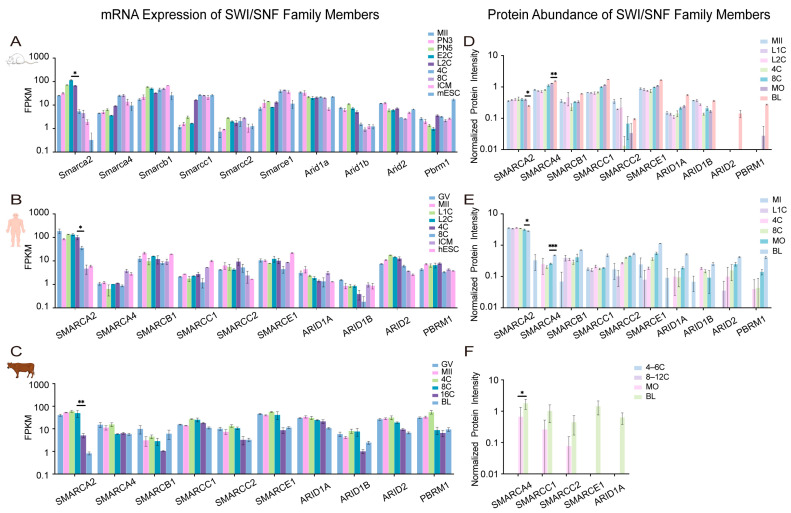
The expression of SWI/SNF family members in embryonic development across different species. (**A**–**C**) The transcript expression of SWI/SNF family members in the early embryonic development of mouse, human and bovine. (**D**–**F**) Protein expression of SWI/SNF family members in the early embryonic development of mouse, human and bovine. GV, oocytes at germinal vesicle; MI, metaphase I; MII, metaphase II; PN3, pronuclear stage 3; PN5, pronuclear stage 5; L1C, late 1-cell embryo; E2C, early 2-cell embryo; L2C, late 2-cell embryo; 4C, 4-cell embryo; 6C, 6-cell embryo; 8C, 8-cell embryo; 12C, 12-cell embryo; 16C, 16-cell embryo; MO, morula; ICM, inner cell mass; hESC, human embryonic stem cell; mESC, mouse embryonic stem cell; BL, blastocyst. Data are plotted on a log_10_ scale to visualize the wide expression range. Statistical analysis was performed using the unpaired two-tailed Student’s *t*-test for comparisons between two adjacent groups. Data are presented as the mean ± SEM. * *p* < 0.05, ** *p* < 0.01, *** *p* < 0.001. Inset panels display low-abundance subunits using an adjusted local *y*-axis scale.

**Figure 5:**
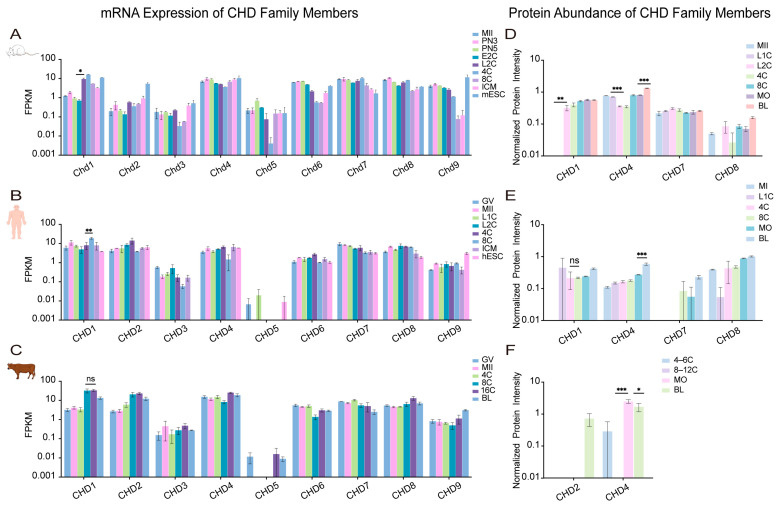
The expression of CHD family members in embryonic development across different species. (**A**–**C**) The transcript expression of CHD family members in the early embryonic development of mouse, human and bovine. (**D**–**F**) Protein expression of CHD family members in the early embryonic development of mouse, human and bovine. GV, oocytes at germinal vesicle; MI, metaphase I; MII, metaphase II; PN3, pronuclear stage 3; PN5, pronuclear stage 5; L1C, late 1-cell embryo; E2C, early 2-cell embryo; L2C, late 2-cell embryo; 4C, 4-cell embryo; 6C, 6-cell embryo; 8C, 8-cell embryo; 12C, 12-cell embryo; 16C, 16-cell embryo; MO, morula; ICM, inner cell mass; hESC, human embryonic stem cell; mESC, mouse embryonic stem cell; BL, blastocyst. Data are plotted on a log_10_ scale to visualize the wide expression range. Statistical analysis was performed using the unpaired two-tailed Student’s *t*-test for comparisons between two adjacent groups. Data are presented as the mean ± SEM. * *p* < 0.05, ** *p* < 0.01, *** *p* < 0.001, ns (not significant). Inset panels display low-abundance subunits using an adjusted local *y*-axis scale.

**Figure 6:**
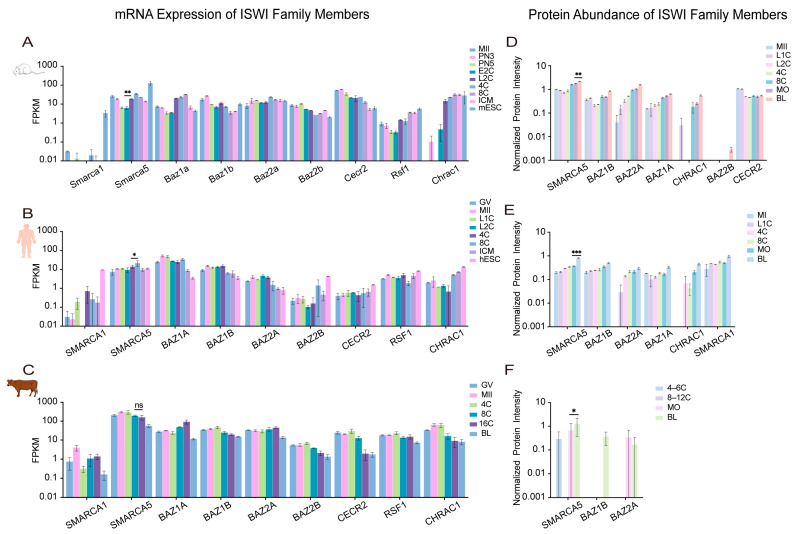
The expression of ISWI family members in embryonic development across different species. (**A**–**C**) The transcript expression of ISWI family members in the early embryonic development of mouse, human and bovine. (**D**–**F**) Protein expression of ISWI family members in the early embryonic development of mouse, human and bovine. GV, oocytes at germinal vesicle; MI, metaphase I; MII, metaphase II; PN3, pronuclear stage 3; PN5, pronuclear stage 5; L1C, late 1-cell embryo; E2C, early 2-cell embryo; L2C, late 2-cell embryo; 4C, 4-cell embryo; 6C, 6-cell embryo; 8C, 8-cell embryo; 12C, 12-cell embryo; 16C, 16-cell embryo; MO, morula; ICM, inner cell mass; hESC, human embryonic stem cell; mESC, mouse embryonic stem cell; BL, blastocyst. Data are plotted on a log_10_ scale to visualize the wide expression range. Statistical analysis was performed using the unpaired two-tailed Student’s *t*-test for comparisons between two adjacent groups. Data are presented as the mean ± SEM. * *p* < 0.05, ** *p* < 0.01, *** *p* < 0.001; ns, not significant. Inset panels display low-abundance subunits using an adjusted local scale.

**Figure 7:**
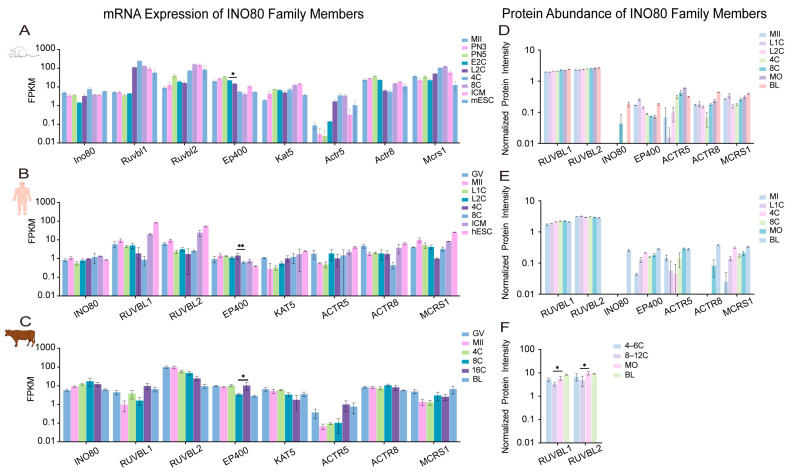
The expression of INO80 family members in embryonic development across different species. (**A**–**C**) The transcript expression of INO80 family members in the early embryonic development of mouse, human and bovine. (**D**–**F**) Protein expression of INO80 family members in the early embryonic development of mouse, human and bovine. GV, oocytes at germinal vesicle; MI, metaphase I; MII, metaphase II; PN3, pronuclear stage 3; PN5, pronuclear stage 5; L1C, late 1-cell embryo; E2C, early 2-cell embryo; L2C, late 2-cell embryo; 4C, 4-cell embryo; 6C, 6-cell embryo; 8C, 8-cell embryo; 12C, 12-cell embryo; 16C, 16-cell embryo; MO, morula; ICM, inner cell mass; hESC, human embryonic stem cell; mESC, mouse embryonic stem cell; BL, blastocyst. Data are plotted on a log_10_ scale to visualize the wide expression range. Statistical analysis was performed using the unpaired two-tailed Student’s *t*-test for comparisons between two adjacent groups. Data are presented as the mean ± SEM. * *p* < 0.05, ** *p* < 0.01. Inset panels display low-abundance subunits using an adjusted local *y*-axis scale.

To independently support the mechanistic roles of chromatin remodelers, we reanalyzed publicly available transcriptomic datasets from humans [109], cattle [79], and mice [110]. For clarity, gene symbols are used according to species-specific convention: human and bovine symbols are in all uppercase (e.g., *SMARCA2*), while mouse symbols have only the first letter capitalized (e.g., *Smarca2*). Our analysis focused on the conserved temporal expression dynamics of each factor across development. The stage-specific activation or repression of these remodelers, detailed below, aligns with key developmental transitions and provides transcriptional support for the hypothesis of their functional coordination [8] (Figure 4, Figure 5, Figure 6 and Figure 7).

Across species, each remodeling family exhibited distinct yet functionally coherent expression trajectories. SWI/SNF ATPases, particularly *SMARCA2*/*Smarca2*, were highly expressed from the maternal stage but declined sharply upon EGA (Figure 4A–C), consistent with their role in chromatin priming rather than transcriptional execution [14,81]. In contrast, a conserved upregulation of *CHD1*/*Chd1* (CHD) transcripts during EGA was observed across all three species (Figure 5A–C), aligning with its essential role in histone deposition for genome activation [72,73]. The ISWI and INO80-related families displayed more complex, species-divergent dynamics: *SMARCA5*/*Smarca5* (ISWI) rose significantly during EGA in human and mouse embryos (Figure 6A,C), while *EP400*/*Ep400* (INO80-related) was induced in human and mouse but declined in bovine embryos (Figure 7A–C). This pronounced species divergence, particularly within the INO80 family, suggests adaptive specialization in chromatin resetting mechanisms across mammals [76,77].

Together, these cross-species transcriptomic profiles indicate that the timing of remodeler activation consistently parallels key developmental transitions, including genome activation, early lineage segregation, and pluripotency establishment. While expression dynamics alone do not establish causality, these patterns provide a transcriptional context that is concordant with experimentally defined functions reported in the literature.

### 4.2. Proteomic Dynamics Highlight Functional Dosage, Temporal Coordination, and Post-Transcriptional Regulation

To complement RNA-level analysis, we integrated proteomic evidence from human [111], bovine [112], and mouse [111] embryos, enabling direct assessment of protein-level abundance patterns that may relate to functional dosage, though not necessarily indicating enzymatic activity at specific loci (Figure 4, Figure 5, Figure 6 and Figure 7).

Protein-level dynamics broadly paralleled transcriptomic trends but also revealed family-specific post-transcriptional regulation. In the SWI/SNF family, proteomic data corroborated the known species-specific functional dominance [14,81], with SMARCA4 as the principal ATPase in human and bovine embryos, and SMARCA2 prevailing in mice (Figure 4D–F). CHD family dynamics revealed both conserved and species-specific regulation: CHD1 protein levels remained stable during human and mouse EGA despite fluctuating transcripts (Figure 5D,F), suggesting post-transcriptional control. Conversely, CHD4 accumulation during the morula-to-blastocyst transition was observed in human and mouse embryos but not in cattle, where its levels instead declined (Figure 5D–F). This indicates divergent regulatory strategies for this repressive remodeler across species [77,93]. ISWI ATPase SMARCA5 accumulated markedly after the morula stage (Figure 6D–F), and its depletion compromises lineage specification [70]. This suggests that SMARCA5-mediated remodeling is critical for the large-scale chromatin reorganization that underpins lineage maturation, rather than for earlier developmental events. INO80-related subunits RUVBL1/2 were constitutively expressed across species and stages (Figure 7D–F), consistent with their conserved, essential roles as structural and catalytic cores of multiple ATP-dependent remodeling complexes [107].

Taken together, the combined RNA and protein analyses highlight that chromatin remodeler abundance is regulated at multiple levels, including transcription, protein stability, and complex composition, in a manner that is temporally aligned with early developmental transitions.

### 4.3. Inter-Family Coordination, Antagonism, and Division of Labor Revealed by Integrated Multi-Omics Analysis

Integrated analysis of transcriptomic and proteomic data suggests that the four remodeling families may not function as isolated units but instead display partially coordinated expression patterns that shift across developmental stages. This interpretation is based on correlative multi-omics data and will require further experimental validation to establish direct functional relationships.

#### 4.3.1. Cooperative Modules During EGA

Our cross-species data reveal that the conserved transcriptional upregulation of *CHD1* (*Chd1*), *EP400* (*Ep400*), and *SMARCA5* (*Smarca5*) during EGA (Figure 5, Figure 6 and Figure 7) signifies a coordinated preparatory phase. This co-expression suggests a functional sequence wherein the histone variant deposition activities of CHD1 and EP400, which are essential for EGA itself [73,75], are transcriptionally primed alongside the ISWI remodeler SMARCA5. The protein product of SMARCA5 then accumulates post-morula (Figure 6D–F) to execute its defined role in nucleosome spacing for lineage maturation [70]. Based on these temporal expression patterns, it is plausible that CHD1-, EP400-, and SMARCA5-associated activities are deployed in a coordinated manner during EGA. However, direct mechanistic interactions remain to be demonstrated experimentally.

#### 4.3.2. Antagonistic Balance During Lineage Segregation

Following EGA, SWI/SNF ATPases (SMARCA2/4) and the repressive remodeler CHD4 exhibit concurrent rises in expression and protein abundance in specific species (Figure 4 and Figure 5), pointing to a potential antagonistic relationship. SWI/SNF complexes promote lineage-specific enhancer activation [65,89,91], whereas CHD4 restricts ectopic lineage programs to ensure fidelity [77,93]. The complementary dynamics of these factors, which are evident from CHD4 accumulation during lineage maturation in human and mouse embryos but not in cattle (Figure 5D–F), are consistent with these established activating and repressive functions, and highlight species- and stage-dependent differences in the deployment of these activities.

#### 4.3.3. Division of Labor Guided by Temporal and Structural Specificity

The distinct temporal expression and accumulation patterns of remodelers, as detailed in our transcriptomic and proteomic atlases (Figure 4, Figure 5, Figure 6 and Figure 7), suggest non-overlapping enrichments among the four remodeling families, though a strict division of labor has not been conclusively established. SWI/SNF complexes are enriched early (Figure 4), a period that correlates with their role in initial chromatin opening [14,81]. CHD family members, notably CHD1, maintain stable protein levels during EGA (Figure 5D,F), a finding that is consistent with a role in fine-tuning the transcriptional landscape [72,73]. ISWI ATPase SMARCA5 accumulates markedly after the morula stage (Figure 6D–F), thereby aligning with its requirement for chromatin consolidation during lineage specification [70]. In contrast, INO80-complex subunits (e.g., RUVBL1/2) are constitutively expressed across species and stages (Figure 7D–F), which suggests a foundational, stage-independent role in maintaining chromatin homeostasis [107].

Collectively, these stage-enriched expression and accumulation patterns of different remodeling families are consistent with previously reported roles in chromatin opening, nucleosome organization, and transcriptional regulation [8]. However, this “division of labor” should be viewed as a hypothesis derived from correlative multi-omics trends rather than as evidence of discrete mechanistic partitioning. Future perturbation-based studies will be required to substantiate or refine this framework.

## 5. Chromatin Remodeling Factors in Non-Mammalian Embryogenesis

### 5.1. Developmental Context of Non-Mammalian Models

In non-mammalian species such as *Xenopus laevis*, embryogenesis proceeds from the zygote through blastula, gastrula, neurula, and tail-bud stages, ultimately establishing the three germ layers and a complex body plan [113,114]. Early cleavage divisions and blastula formation are followed by region-specific cellular differentiation, coordinated cell migration during gastrulation, and neural tube formation during neurulation, culminating in tissue- and organ-specific differentiation.

### 5.2. Key Roles in Axis Patterning and Germ Layer Segregation

Although studies of chromatin remodeling factors during the earliest stages of non-mammalian embryogenesis remain limited, accumulating evidence highlights their critical roles in gastrulation and neural development. In *Xenopus*, knockdown of *brg1* arrests embryos at the blastula or early gastrula stage, demonstrating that Brg1 amplifies transcriptional bursts to elevate key developmental regulators, including BCNE/Nieuwkoop center genes (e.g., *chordin*, *noggin*, *hhex*, *cer*) and BMP-dependent ventral genes (*vent1*, *vent2*), thereby orchestrating dorsoventral axis formation and organizer activity [115]. Chd4 also plays an essential role in germ layer patterning: by repressing the transcription factor Smad interacting protein 1 (Sip1, a member of the zinc finger E-box binding homeobox 1 family, ZEB1), Chd4 modulates the sensitivity of the *Xenopus* Brachyury (*Xbra*) promoter to Activin/Nodal signaling, regulating the boundary between neuroectoderm and mesoderm [116,117,118].

Collectively, these findings reveal that chromatin remodeling factors govern transcriptional dynamics and spatial patterning during non-mammalian embryogenesis. The mechanistic insights gained from models such as *Xenopus* establish an essential foundation for exploring the conserved and divergent functions of these remodelers across vertebrate species, thereby complementing and enriching findings from mammalian embryonic stem cell studies.

## 6. Conclusions and Perspectives

**Figure 8:**
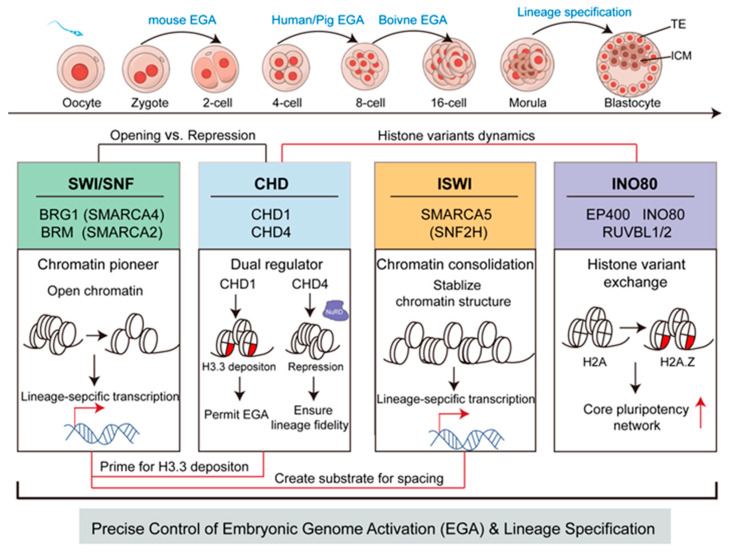
A coordinated network of chromatin remodelers in early mammalian embryogenesis. This schematic provides a conceptual, hypothesis-generating model that integrates cross-species multi-omics evidence to illustrate the proposed functional interactions among the four major remodeling families. Connecting lines represent inferred relationships: cooperation (black lines) and antagonism (red lines). The spatial arrangement of the families reflects a proposed temporal “division of labor”, with SWI/SNF acting as a chromatin pioneer, CHD providing dual fine-tuning, ISWI consolidating nucleosome architecture, and INO80 maintaining dynamics through histone variant exchange. Red arrows represent transcriptional activation. These interpretations are derived from correlative trends and await direct experimental validation.

Early mammalian embryogenesis requires the coordinated action of multiple ATP-dependent chromatin remodeling families, whose functions cannot be understood in isolation. By integrating functional studies with cross-species transcriptomic and proteomic analyses, this review highlights how SWI/SNF, ISWI, CHD, and INO80 complexes are differentially engaged across developmental transitions to support embryonic genome activation, lineage specification, and stabilization of cell fate programs. Rather than defining a rigid mechanistic hierarchy, the conceptual framework summarized in Figure 8 reflects an evidence-based synthesis of when and where distinct remodeling activities are deployed during early development.

A major challenge moving forward is to resolve how chromatin remodeling activities are coordinated at specific genomic loci and within higher-order chromatin structures. Recent advances in low-input and single-cell chromatin profiling approaches, including ATAC-seq, CUT&RUN/CUT&Tag, and three-dimensional genome mapping methods such as Hi-C, provide opportunities to directly link remodeler occupancy and activity to nucleosome positioning, enhancer–promoter communication, and transcriptional output during defined developmental windows [119,120,121,122,123,124]. Applying these technologies in perturbation-based and cross-species contexts will be essential to move from correlative patterns toward causal mechanisms.

From a translational perspective, understanding how chromatin remodeling factors govern early developmental decisions has direct implications for human disease and regenerative medicine. The frequent involvement of SWI/SNF, CHD, and INO80 subunits in congenital disorders and cancer underscores the developmental origins of these pathologies [12,13,14,15,27,29,51,125], while emerging reprogramming and stem-cell studies suggest that targeted modulation of remodeling activity may offer new strategies for controlling cell fate [72,73,81,83,107]. Together, these perspectives position chromatin remodelers as central regulators linking early embryogenesis, disease etiology, and therapeutic intervention.

## 7. Limitations

Although this review integrates cross-species transcriptomic, proteomic, and functional evidence, several limitations must be acknowledged. First, the majority of mechanistic studies on ATP-dependent chromatin remodelers come from a limited number of vertebrate species, such as mouse, cattle, pig, and Xenopus. As a result, the extent to which these conclusions can be generalized across all vertebrates remains uncertain. Functional redundancy among remodeler subunits and species-specific developmental timing also complicates interpretation of cross-species comparisons.

Second, our multi-omics analysis is inherently correlative. Neither transcript abundance nor protein levels directly reflect remodeling activity, which is strongly influenced by complex assembly, chromatin context, post-translational modifications, and subcellular localization. Consequently, the temporal patterns we describe should be interpreted as hypothesis-generating rather than causal evidence of remodeler function. Moreover, current low-input proteomic technologies still have limited sensitivity for low-abundance subunits, potentially leading to an underestimation of their developmental contribution.

Finally, the conceptual framework proposed here is intended as an integrative model rather than a mechanistic conclusion. It emphasizes potential cooperation, antagonism, and a temporal division of labor among remodeling families. Direct evidence for inter-family communication, locus-specific nucleosome positioning, or three-dimensional chromatin restructuring remains sparse. Future perturbation-based and single-cell multi-omics studies will thus be essential to test and refine these proposed relationships.

## Figures and Tables

**Table 1:** Roles of Chromatin Remodeling Factors on Embryonic Development Across Species.

Complex	Member	Species/Cell	Key Events
SWI/SNF	SMARCA4	mice	EGA/lineage specification [65,66]
	ARID1A	porcine	EGA [13]
	ARID1A	mice	germ-layer formation [67]
	SMARCC1	mice	vascular and cardiac morphogenesis [68]
	SMARCC1	mice	lineage specification [69]
ISWI	SMARCA5	mice/cattle	lineage specification [70]
	SMARCA2	porcine	EGA to blastocyst stage [71]
CHD	CHD1	mice/cattle	EGA/lineage specification [72,73]
	CHD4	mice	lineage specification [74]
INO80	EP400	mice	EGA [75]
	INO80	porcine	lineage specification [76]
	MCRS1	mice	lineage specification [77]

## Data Availability

No new data were created or analyzed in this study. Data sharing is not applicable to this article.
